# Supplementary material for: Development and Determinants of Topsoil Bacterial and Fungal Communities of Afforestation by Aerial Sowing in Tengger Desert, China
Source: J Fungi (Basel). 2023 Mar 24;9(4):399. doi: 10.3390/jof9040399 (PMC10144444; doi:10.3390/jof9040399)
Supplement: Supplementary file 1 [file jof-09-00399-s001.zip › jof-2285618-supplementary.pdf]

Table S1. The surface vegetation cover and morphological characteristics of sampling sites.

Sampling sites	Species of air-seeding	Composition of shrub layer /%	Height of shrub (Mean±S.D.) /cm	Coverage of shrubs (Mean±S.D.) /%	Coverage of herbs (Mean±S.D.) /%	Geomorphic types	Elevation (Mean±S.D.) /m	Landscape photos
A0	None	None	None	None	2.3±1.6	Semifixed dune	1299±4	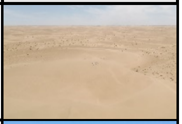
A3	*A.d.* + *C.s.* + *C.m.*	56:26:18	68.6±37.2	4.5±2.7	1.2±1.4	Semifixed dune	1311±6	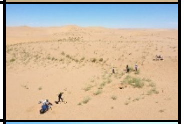
A5	*A.d.*	100	46.3±14.2	12.5±0.3	6.3±2.7	Fixed dune	1306±4	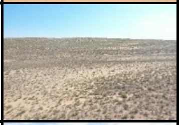
A11	*A.d.* + *C.m.*	72:38	57.0±17.7	12.8±1.3	4.3±3.0	Fixed dune	1292±7	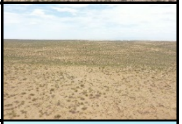
A20	*A.d.* + *C.s.* + *C.m.*	46:41:13	76.4±45.0	11.1±3.7	12.2±6.9	Fixed dune	1368±14	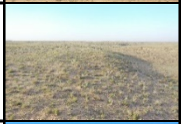
A29	*A.d.* + *C.s.* + *C.m.*	10:35:55	93.1±52.1	10.4±5.7	10.9±5.9	Fixed dune	1261±12	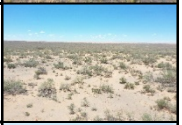
A37	*A.d.* + *C.s.* + *C.m.*	58:39:3	85.8±71.3	14.2±5.2	22.5±10.5	Fixed dune	1432±2	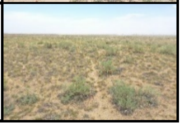

Abbreviation of species: C. m., Calligonum mongolicum Turcz.; C. s., Corethrodendron scoparium Fisch. et Basiner; A. d., Artemisia desertorum Spreng.

Table S2. Changes of soil physicochemical properties at two depths along the stages of afforestation by aerial seeding in the northeastern of Tengger Desert, China. The multivariate analysis of covariance for stages, depths, and stages × depths interaction for soil physicochemical properties was also presented in behind.

Depths (cm)	Stages	pH	EC (μS/cm)	Na (mg/L)	K (mg/L)	Mg (mg/L)	Ca (mg/L)	TC (g/kg)	SOC (g/kg)	TN (g/kg)	TP (g/kg)	AP (mg/kg)	AK (mg/kg)
0~10	A0	8.88^{a}	22.16^{c}	2.77^{bc}	1.28^{b}	0.53^{b}	10.87^{bc}	1.40^{b}	0.67^{ab}	0.30^{ab}	0.13^{a}	1.35^{ab}	72.74^{a}
	A3	9.02^{a}	33.90^{bc}	8.03^{a}	1.89^{b}	0.83^{ab}	8.70^{c}	1.91^{b}	0.59^{b}	0.28^{b}	0.14^{a}	0.78^{b}	58.28^{a}
	A5	9.06^{a}	45.04^{abc}	6.16^{ab}	4.84^{a}	1.09^{ab}	25.90^{ab}	2.75^{a}	1.09^{ab}	0.32^{ab}	0.14^{a}	0.98^{ab}	77.70^{a}
	A11	9.07^{a}	53.10^{ab}	5.24^{abc}	3.73^{a}	0.90^{ab}	21.08^{abc}	3.22^{a}	1.01^{ab}	0.33^{ab}	0.14^{a}	1.38^{ab}	86.99^{a}
	A20	9.12^{a}	57.80^{a}	2.24^{b}	4.96^{a}	1.23^{ab}	32.05^{a}	3.03^{a}	1.30^{a}	0.35^{ab}	0.13^{a}	2.69^{a}	69.63^{a}
	A29	8.99^{a}	46.24^{ab}	6.49^{ab}	4.55^{a}	1.48^{a}	21.24^{abc}	2.97^{a}	0.79^{ab}	0.31^{ab}	0.12^{a}	0.97^{ab}	63.68^{a}
	A37	9.06^{a}	55.24^{a}	4.55^{abc}	4.77^{a}	1.12^{ab}	24.87^{ab}	3.02^{a}	1.12^{ab}	0.35^{a}	0.12^{a}	2.37^{ab}	60.31^{a}
10~20	A0	8.82^{a}	18.60^{c}	3.78^{cd}	1.80^{ab}	0.60^{bc}	7.68^{bc}	1.44^{c}	0.70^{ab}	0.28^{b}	0.12^{abc}	1.01^{ab}	69.82^{ab}
	A3	8.97^{a}	31.06^{bc}	7.67^{ab}	1.60^{b}	0.72^{c}	7.27^{c}	1.84^{bc}	0.55^{b}	0.30^{b}	0.14^{ab}	0.96^{ab}	54.35^{ab}
	A5	9.14^{a}	55.92^{a}	9.24^{a}	3.77^{ab}	1.68^{ab}	33.13^{a}	2.68^{a}	1.08^{a}	0.31^{b}	0.15^{a}	0.66^{b}	69.83^{a}
	A11	9.21^{a}	49.82^{ab}	4.72^{bcd}	2.31^{ab}	0.84^{abc}	25.07^{a}	3.08^{a}	0.96^{ab}	0.32^{ab}	0.14^{ab}	1.31^{ab}	65.09^{ab}
	A20	9.16^{a}	53.32^{a}	2.75^{abc}	1.81^{b}	1.69^{a}	34.35^{a}	2.74^{a}	0.91^{ab}	0.32^{ab}	0.13^{abc}	1.53^{a}	44.35^{b}
	A29	9.17^{a}	44.32^{ab}	6.39^{d}	2.75^{ab}	1.55^{abc}	21.50^{ab}	2.66^{ab}	0.64^{b}	0.29^{b}	0.11^{bc}	0.50^{b}	49.35^{ab}
	A37	9.26^{a}	47.26^{ab}	3.60^{cd}	4.48^{a}	1.27^{abc}	24.78^{a}	2.72^{a}	0.78^{ab}	0.35^{a}	0.10^{c}	0.64^{b}	44.58^{b}
Stages		1.75	13.08^{***}	14.52^{***}	4.98^{***}	6.83^{***}	17.98^{***}	19.99^{***}	7.08^{***}	6.75^{***}	6.30^{***}	4.38^{**}	5.19^{**}
Depths		2.23	0.55	0.68	9.28^{**}	2.92	0.91	3.36	5.99^{*}	2.67	1.44	10.21^{**}	17.32^{***}
Stages× Depths		0.48	0.93	1.56	1.32	0.97	0.61	0.27	1.08	0.62	0.36	2.05	0.94

Values are mean of 5 replicate sampling sites, and the significance of difference among stages were represented by superscript letters at $P < 0.05$. Significance level, * $p < 0.05$, ** $p < 0.01$, *** $p < 0.001$.

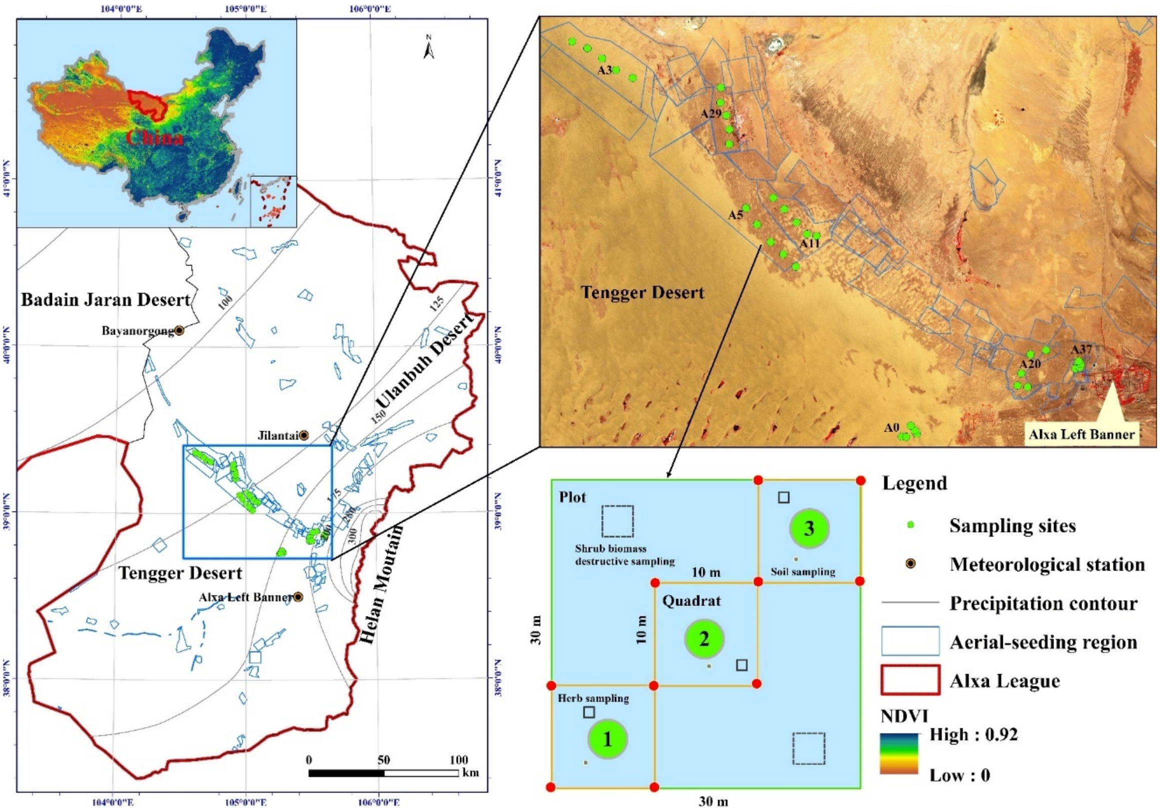

Figure S1. Sketch of the study area and sampling sites setting.

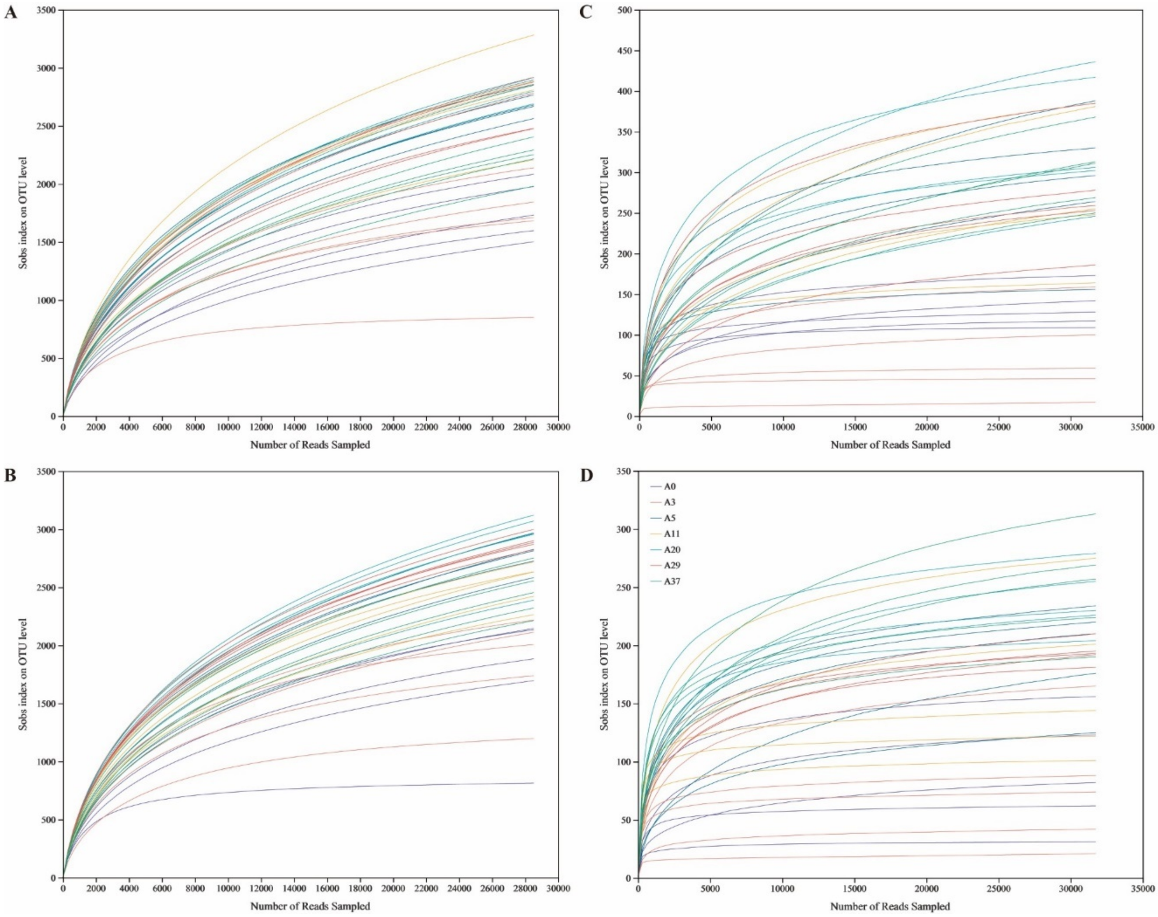

Figure S2. Rarefaction curves of bacterial community (A, 0 ~ 10 cm; B, 10 ~ 20 cm) and fungal community (C, 0 ~ 10 cm; D, 10 ~ 20 cm) for different stages of afforestation by aerial seeding in the northeastern of Tengger Desert, China.

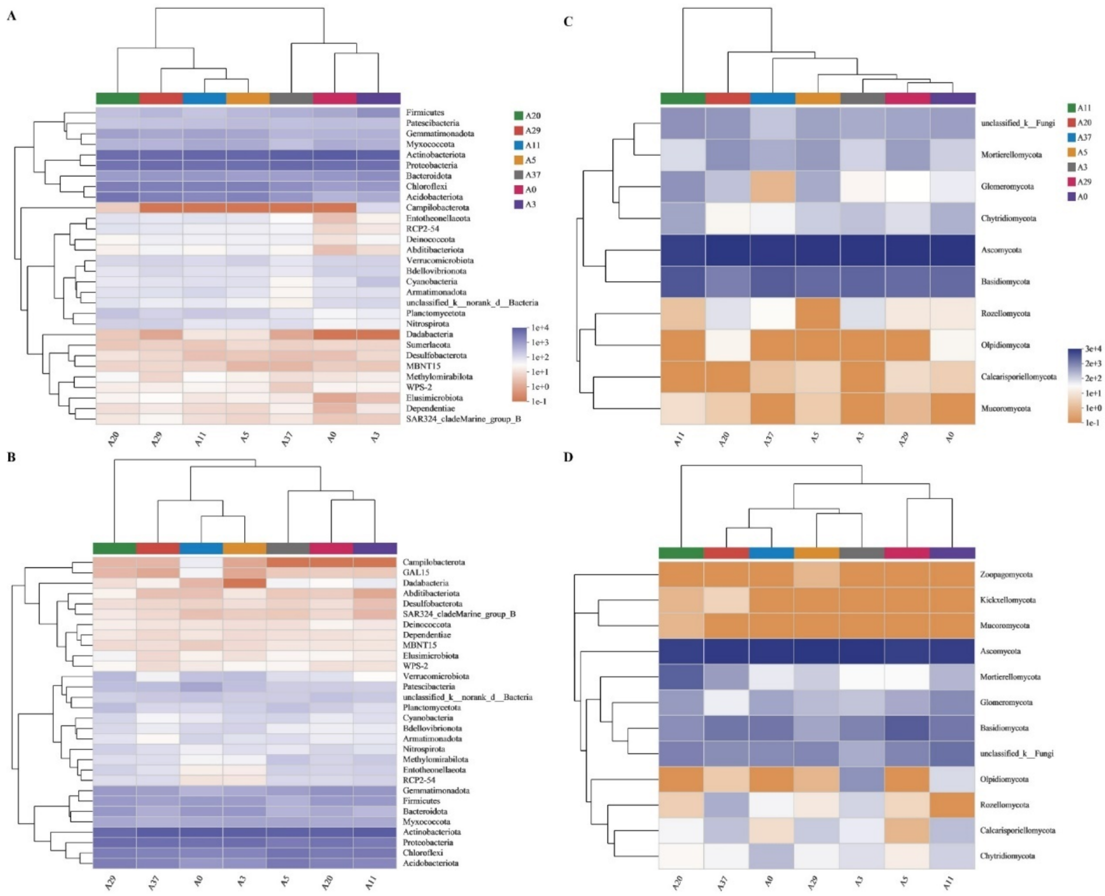

Figure S3. Heatmap of dominant phylum bacterial community (A, 0 ~ 10 cm; B, 10 ~ 20 cm) and fungal community (C, 0 ~ 10 cm; D, 10 ~ 20 cm) for different stages of afforestation by aerial seeding in the northeastern of Tengger Desert, China. The bacterial community was the top 30 phyla and fungal community for all phyla.
